# Global and regional prevalence, burden, and risk factors for MASLD in children and adolescents aged 5 to 24 years: a systematic review, meta-analysis, and modeling study

**DOI:** 10.1186/s12916-026-04801-3

**Published:** 2026-03-18

**Authors:** Yunfei Liu, Shan Cai, Ruolan Yang, Junkang Lin, Jiajia Dang, Tianyu Huang, Jiaxin Li, Kaiheng Zhu, Ziyue Chen, Yihang Zhang, Yi Song, Susan M. Sawyer

**Affiliations:** 1 Institute of Child and Adolescent Health, School of Public Health, National Health Commission Key Laboratory of Reproductive Health, Peking University, Beijing, China; 2 Department of Paediatrics, Faculty of Medicine, Dentistry and Health Sciences, The University of Melbourne, Parkville, VIC 3010 Australia; 3 Murdoch Children’s Research Institute, Parkville, VIC 3052 Australia; 4 Centre for Adolescent Health, Royal Children’s Hospital, Parkville, VIC 3052 Australia

**Keywords:** Metabolic dysfunction-associated steatotic liver disease, Children and adolescents, Meta-analysis, Prevalence, Obesity

## Abstract

**Background:**

Obesity is associated with metabolic dysfunction-associated steatotic liver disease (MASLD). Despite the rising prevalence of obesity among children and adolescents, no studies have examined risk factors or developed models to estimate MASLD burden by sex, age group, or geographic location.

**Objective:**

To estimate and predict the distribution and shifting patterns of the burden of MASLD in children and adolescents aged 5 to 24 years, globally, regionally, and in China.

**Methods:**

We systematically searched PubMed, EMBASE, Web of Science, Cochrane, and CNKI for studies reporting the prevalence of MASLD and its closely related diagnostic constructs, including metabolic dysfunction-associated fatty liver disease (MAFLD) and non-alcoholic fatty liver disease (NAFLD), in 5–24-year-olds, and synthesized evidence across these definitions to estimate the burden of MASLD. Random-effects meta-regression synthesized age-, sex-, and year-specific prevalence and risk factors. Additionally, using data from the Global Burden of Disease, World Population Prospects, and Chinese National Survey on Students’ Constitution and Health, a risk factor-based model estimated global, regional, and provincial (China) MASLD burden. The protocol was registered in PROSPERO (CRD420251062351).

**Results:**

Of 2747 records, 56 studies (54 English, 2 Chinese) were included; 37 informed prevalence and 38 informed risk factors. Our model indicated that the global MASLD prevalence among 5–24-year-olds was 7.0% (95% CI: 4.1, 11.7), increasing with age and year, and higher in boys. Asia had the largest number of cases in 2000 (63.8 million [51.5, 76.6]) and 2020 (160.9 million [134.5, 187.6]). Our model further indicated that Africa is projected to surpass Asia in total case numbers from 2040 onward. In China, MASLD prevalence among 6–18-year-olds was highest in Hebei, Shandong, and Beijing (2000) and in Tianjin, Shandong, and Heilongjiang (from 2020 onwards).

**Conclusions:**

The prevalence of MASLD among children and adolescents continues to rise alongside the epidemic of obesity. Model-based estimates suggest that the burden of MASLD may shift over time towards currently less developed regions of the world, such as Africa, and less well-developed regions in China. Targeted investment in obesity prevention is urgently needed, as are health services to reduce the health impacts of MASLD during and beyond childhood and adolescence.

**Supplementary Information:**

The online version contains supplementary material available at 10.1186/s12916-026-04801-3.

## Background

The rapid global rise in obesity prevalence has led to a series of health problems, including diabetes, cardiovascular diseases, and liver disease [1]. Known previously as nonalcoholic fatty liver disease (NAFLD), metabolic dysfunction-associated steatotic liver disease (MASLD) is a leading cause of chronic liver disease and cirrhosis, affecting more than a third of the adult population and approximately 13% of children and adolescents globally [2, 3]. Among children and adolescents with obesity, the prevalence of MASLD is estimated to be at least threefold higher than in the general population [4]. Children and adolescents with MASLD experience higher risks of type 2 diabetes and cardiovascular diseases than the general population [5]. Importantly, these risks are higher than in those with obesity without MASLD [6, 7]. Young people also experience more severe steatosis than adults [8], and a much higher mortality rate than age-sex-matched peers [5]. While MASLD can resolve with weight loss [9], nearly two-thirds of children and adolescents with MASLD have disease that persists into adulthood which is neither diagnosed nor managed [10].

A recent research priority-setting exercise highlighted the need for better data to define the burden of MASLD [11]. However, since the diagnosis of MASLD requires liver biopsy or imaging techniques such as ultrasound and FibroScan [12], it is challenging to obtain these data from large populations, especially in low-and middle-income countries, many of which have a high or growing burden of obesity yet limited health resources for MASLD screening. Global Burden of Disease (GBD) studies provide valuable evidence. However, while fluctuating, the estimated prevalence of MASLD among Chinese adolescents aged 15–24 has reportedly changed little over the past three decades [13]. This limited temporal variation is likely driven by the heavy reliance of GBD estimates on Spatiotemporal Gaussian Process Regression (ST-GPR), which borrow strength across time, age and geography, but that may also attenuate true temporal and regional variation [14]. As a result, such estimates may not reflect the true burden in China, as MASLD is highly prevalent in overweight or obese (OWOB) children and adolescents [15] and OWOB has greatly risen over the past three decades in China, as it has elsewhere [16]. Previous meta-analyses have estimated the global prevalence of MASLD in children and adolescents [4]. However, there has been little in-depth examination of regional variations, which could potentially aid the development of context-specific strategies.

We set out to fill this research gap by conducting a systematic review, meta-analysis, and modeling study using population studies that reported the prevalence of MASLD in children and adolescents aged 5 to 24 years. Children and adolescents were defined as individuals aged 5–24 years, in accordance with contemporary life-course frameworks that conceptualize adolescence as extending into the mid-twenties due to prolonged biological, psychological, and social transitions from childhood to adulthood [17]. We aimed to (1) estimate the global prevalence of MASLD among 5–24-year-olds, and by age group (5–9, 10–14, 15–19, 20–24 years) and sex; (2) establish the main risk factors for MASLD; (3) estimate the regional number of children and adolescents affected with MASLD between 2000 and 2020, and the predicted number from 2030 to 2050; and (4) estimate the provincial prevalence of MASLD in China between 2000 and 2050.

## Methods

This study consisted of three stages. First, we undertook a systematic review and meta-analysis to synthesize the global and age-sex-year-specific prevalence of MASLD among children and adolescents aged 5 to 24 years, and to explore the risk factors for MASLD. Next, we used data on OWOB among children and adolescents from GBD 2021 and population data from the World Population Prospects to estimate the number of MASLD cases by global region. Finally, China was selected as a case study, in which we used data from the Chinese National Survey on Students’ Constitution and Health (CNSSCH) to estimate provincial-level prevalence. The protocol for this study is available at PROSPERO (protocol number CRD420251062351).

### Search strategy and selection criteria

Two investigators (YL and RY) independently led a comprehensive literature search in five bibliographic databases (PubMed, EMBASE, Web of Science, Cochrane, and China National Knowledge Infrastructure (CNKI)) to identify all studies (without restriction by year) that have reported the prevalence of MASLD and its closely related diagnostic constructs, including metabolic dysfunction-associated fatty liver disease (MAFLD) and NAFLD, in 5–24-year-olds. These definitions represent overlapping clinical constructs characterized by hepatic steatosis and share largely similar target populations. Previous studies have shown that 99.8% of individuals with NAFLD meet the diagnostic criteria for MASLD, supporting the substantial overlap between these definitions [18]. Given the historical evolution of disease nomenclature and the limited availability of population-based MASLD-specific data, evidence from studies using MAFLD and NAFLD definitions was synthesized to inform MASLD prevalence estimation.

The literature search was undertaken on 14 August, 2025, and details of the search strategies are presented in Additional file 1: Table S1. After excluding duplicate records, the same investigators independently screened all abstracts. Studies were included if they met the following criteria: (1) population-based design, including those targeting the general population or children and adolescents with overweight/obese; (2) reported the prevalence of MASLD, MAFLD, or NAFLD; (3) included children or adolescents aged 5 to 24 years as all or part of the study population; and (4) were published in English or Chinese. The exclusion criteria were (1) GBD [19] or case–control studies were excluded because they do not provide directly observed, population-representative prevalence estimates; (2) conference abstracts, review articles, or case reports were excluded; (3) studies conducted in populations that were not representative of the general population (e.g., individuals with comorbid conditions such as type 2 diabetes) were excluded; and (4) studies lacking clear information on the age of participants were excluded.

### Data extraction and quality assessment

From papers that met these criteria, three investigators (YL, RY, and JL) independently extracted the following information: title, first author, publication year, investigation year, study location, study design, population (general population, overweight population, obese population), sampling strategy, diagnostic criteria, sample size, number of cases, proportion of girls, and age. Each study was reviewed by two reviewers. When the investigation year was missing, we imputed it using the average difference between the investigation year and the publication year (5 years). For those studies with exploration of risk factors associated with MASLD, we extracted the definition of factors, with the corresponding odds ratio (OR) and 95% confidence interval (CI).

Three investigators (YL, RY, and JL) independently assessed study quality according to the Strengthening the Reporting of Observational Studies in Epidemiology (STROBE) with five dimensions, including sample population, sample size, participation rate, outcome assessment, and analytical methods [20]. All discrepancies in data extraction and quality assessment were resolved through discussion and consensus.

### Data sources

#### Global Burden of Disease 2021

GBD 2021 produced the estimates of incidence, prevalence, disability-adjusted life-years (DALYs), and healthy life expectancy (HALE) for 371 diseases and injuries by sex, age, and year in 204 countries and territories. The details of GBD 2021 have been published elsewhere [14]. Building on this work, the prevalence of OWOB was estimated, based on International Obesity Task Force (IOTF) criteria for 5–24-year-olds for 204 countries and territories from 1990 to 2021, and with forecasting from 2022 to 2050 [16]. In this study, we utilized the country-specific prevalence of OWOB stratified by sex and age group from 2000 to 2050.

#### World Population Prospects 2024

World Population Prospects is the official United Nations population estimates and projections that provide age-sex-specific estimates from 1950 to 2023 and projections from 2024 to 2100 for 237 countries and territories [21]. In this study, we utilized the age-sex-specific population for 5–24-year-olds from 2000 to 2050, of which the medium variant scenario was chosen for projections after 2024.

#### Chinese National Survey on Students’ Constitution and Health

The CNSSCH is the largest nationally representative survey of school-aged children aged 6–18 years old in China. Starting in 1985 and generally undertaken every 5 years, the details of CNSSCH have been published elsewhere [22]. In brief, a multistage stratified cluster random sampling design was adopted, ensuring that the sample represented regions with varying levels of development across all provinces. To maximize the number of time points available and thereby improve the reliability of projections, minority populations were excluded (for example, data for Tibetan populations were only available after 2005). Accordingly, this study utilized data on Han Chinese children and adolescents in 29 provinces (Xizang, Hong Kong, Macao, and Taiwan were not included) across seven waves (1985, 1995, 2000, 2005, 2010, 2014, and 2019, the most recently available data). Data were not available in Hainan and Chongqing in 1985, or in Qinghai and Chongqing in 1995). IOTF criteria were used to classify children and adolescents into 3 mutually exclusive categories: non-overweight, overweight, and obese [23]. Informed consent was obtained from all participants and their guardians. This study was approved by the Peking University Biomedical Ethics Committee (No. IRB00001052-19095).

### Primary study variables

The primary study variable was the prevalence and the number of MASLD cases among children and adolescents aged 5–24 years, and also by 5-year age bands where possible. Secondary study variables included a range of risk factors associated with MASLD, including but not limited to body mass index (BMI), sex, age, elevated alanine aminotransferase (ALT), insulin resistance (IR), and triglycerides (TG). These variables were extracted where available and were used in the synthesis of risk factor associations and in subsequent modeling analyses.

### Statistical analysis

#### Synthesis of global and age-specific and sex-specific prevalence of MASLD

To pool the global prevalence of MASLD among 5–24-year-olds, we conducted a multilevel random-effects meta-analysis, using location as the random effect to control the clustering effect of location. Logit transformation was used to stabilize the variance and reduce heteroscedasticity. To assess the robustness of the pooled estimates and potential small-study effects, we conducted influence diagnostics and leave-one-out analyses, and examined funnel plot asymmetry using Egger’s regression test. Given the limited number of MAFLD (*n* = 1) and MASLD (*n* = 2) studies, formal definition-specific subgroup meta-analyses were not feasible.

For the estimation of age-sex-specific prevalence of MASLD, we conducted a multilevel random-effects meta regression, adjusting for the clustering effect of study and location [24], with the following logit link function:

$$logit\left({P}_{ijk}\right)=\mathrm{log}\left(\frac{{P}_{ijk}}{1-{P}_{ijk}}\right)=\alpha +{\beta }_{1}\times age+{\beta }_{2}\times year+{\beta }_{3}\times girl+{u}_{j}+{v}_{k}$$

where ${P}_{ijk}$ represents the prevalence of MASLD for a study or a subset *i* with study *j* and location *k*, *age*, *year*, *and girl* represent the mean age, investigation year, and proportion of girls, ${u}_{j}$ represents the random effect at the location level, and ${v}_{k}$ represents the random effect at the study level. Based on the former equation, we estimated the age-sex-specific MASLD prevalence in 2000, 2010, and 2020.

#### Exploration of risk factors of MASLD

Random effect meta-analysis was used to synthesize the odds ratio (OR) for different risk factors of MASLD among studies that explored the associations using multivariable regression. Risk factors assessed in at least three studies were analyzed overall; those supported by five or more studies were included in the main analysis, whereas factors evaluated in three to five studies were treated as exploratory.

#### Prediction of the age-sex-specific MASLD prevalence in 2030, 2040, and 2050

Due to the strength of available evidence and data availability, BMI was employed for predicting MASLD during 2030 and 2050 and for modeling the regional burden. Using the LMS method [23], we calculated age-sex specific BMI differences between non-overweight populations and populations with overweight or obesity. To align with the GBD data, the 2023 world population obtained from World Population Prospects was applied as weights to estimate age-specific OR for each 5-year age group. The age-sex specific MASLD prevalence in 2030, 2040, and 2050 was predicted using the following equation:

$${P}_{j}={P}_{2020}\times (1+\sum_{RF}({RFprev}_{j}-{RFprev}_{2020})\times ({OR}_{RF}-1))$$

where ${P}_{j}$ represents the MASLD prevalence in 2030, 2040, and 2050; ${RFprev}_{j}$ and ${RFprev}_{2020}$ represent prevalence of risk factors in prediction years and 2020, respectively; ${OR}_{RF}$ represents the OR for each risk factors, namely overweight and obese. We estimated the 95% confidence intervals (CI) through Monte Carlo simulation (10,000 iterations), assuming that the input parameters followed normal distributions.

#### Estimation of the regional burden of MASLD

The synthesized OR and the global age-sex specific prevalence in each year were used to estimate the number of children and adolescents affected with MASLD in five different regions based on the following equation:

$${N}_{region}={Pop}_{region}\times {Prev}_{global}\times (1+\sum_{RF}({RFprev}_{region}-{RFprev}_{global})\times ({OR}_{RF}-1))$$

where ${N}_{region}$ represents the number of MASLD in each region; ${RFprev}_{global}$ and ${RFprev}_{region}$ represent global and regional prevalence of risk factors in each year, respectively; ${OR}_{RF}$ represents the OR for each risk factors. The same Monte Carlo simulation approach (10,000 iterations) was applied here.

#### Estimation of national and provincial prevalence among Chinese 6–18-year-olds

Consistent with the methods used for analyzing and projecting global prevalence, we conducted a subset analysis for the East Asian population and estimated age- and sex-specific prevalence in China from 2000 to 2050, incorporating overweight and obesity prevalence. The synthesized OR and the East Asian and Chinese age-sex specific prevalence in each year were used to estimate the prevalence of MASLD during 2000 to 2050.

$${P}_{China}={P}_{\text{East Asian }}\times (1+\sum_{RF}({RFprev}_{China}-{RFprev}_{\text{East Asian}})\times ({OR}_{RF}-1))$$

where ${P}_{China}$ and ${P}_{\text{East Asian}}$ represents the age-sex specific MASLD prevalence of Chinese and East Asian children and adolescents; ${RFprev}_{China}$ and ${RFprev}_{\text{East Asian}}$ represent prevalence of risk factors for China and East Asian, respectively; ${OR}_{RF}$ represents the OR for each risk factors. The same Monte Carlo simulation approach (10,000 iterations) was applied here.

Based on the CNSSCH, we applied linear regression with normalized weights to project provincial-level prevalence in OWOB for 2020, 2030, 2040, and 2050. Similarly, the above equation was applied to estimate provincial-level prevalence for Chinese 6–18-year-olds.

#### Model assumptions

Our model relies on several key assumptions. First, we assumed that the associations between OWOB and MASLD, as summarized by odds ratios from the literature, are broadly transferrable at the population level across regions and over time. Second, we assumed that changes in OWOB prevalence exert additive and approximately linear effects on MASLD prevalence within the modeled range. In addition, the model does not explicitly account for other factors, such as diet quality, physical activity patterns, or screening and diagnostic practices, which may influence observed MASLD prevalence over time.

#### Sensitivity analysis

In view of the inconsistency in steatosis assessment methods, we performed a sensitivity analysis that was restricted to studies utilizing ultrasound, FibroScan, computed tomography, magnetic resonance imaging, or liver biopsy when pooling data of MASLD prevalence.

All analyses and data visualization were performed with R version 4.2.1. A two-tailed *P* value < 0.05 was considered to be statistically significant.

## Results

### Study selection procedure and characteristics of included studies

**Fig. 1:**
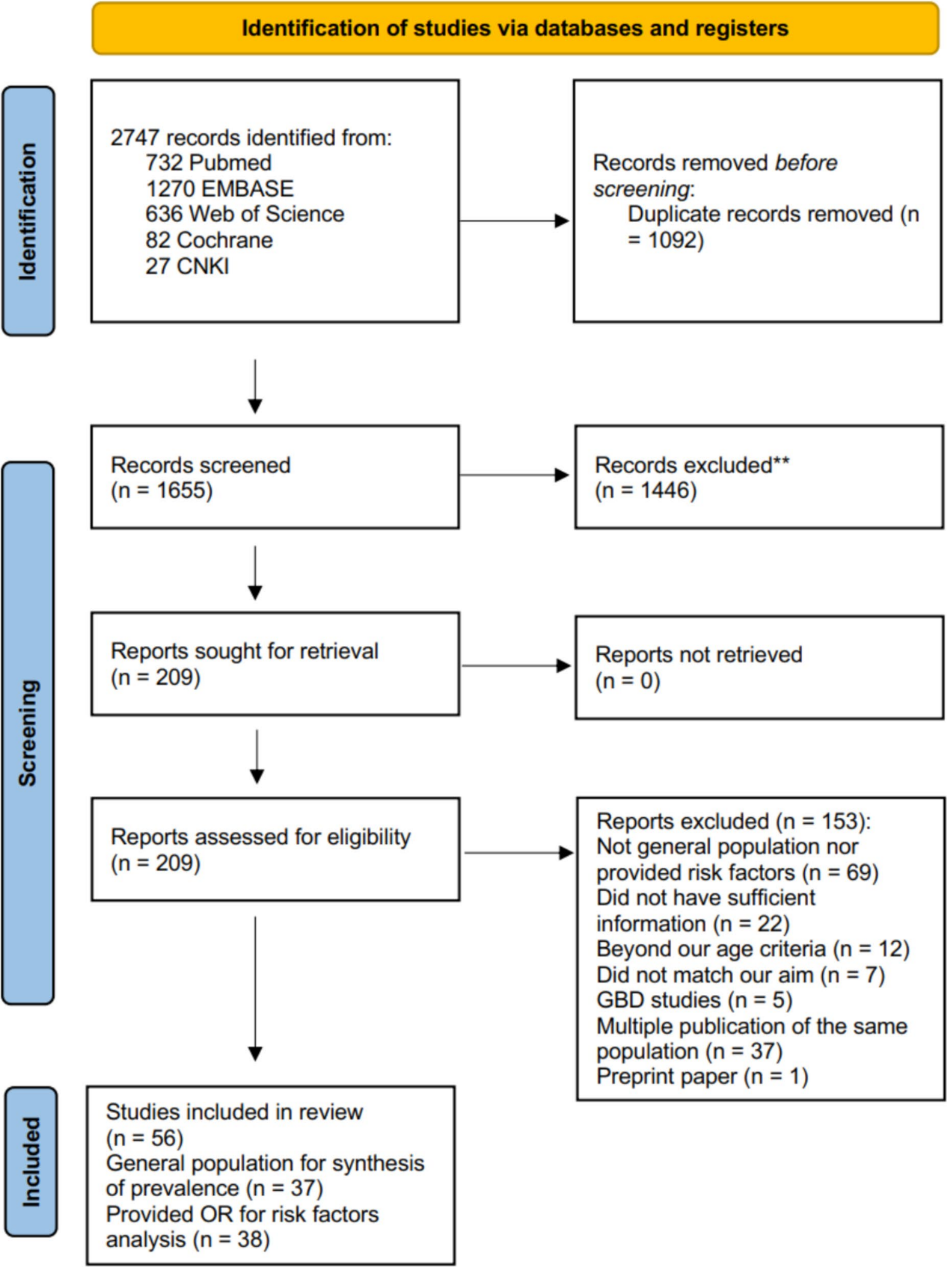
Study search and selection procedure

The initial literature search identified 2747 records. After removing 1092 duplicate records, and excluding 1655 records through title and abstract screening, the full texts of 209 records were reviewed and a further 153 records were excluded. This process resulted in 56 studies (54 in English and 2 in Chinese) included within the analysis, of which 37 were used for synthesizing prevalence and for exploring the relationship between the prevalence of MASLD and age, sex and investigation year. Studies restricted to OWOB populations were excluded from prevalence pooling. In contrast, 38 studies reporting OR for risk factors were all retained for OR synthesis, regardless of whether they were conducted in general populations or in overweight or obese populations (Fig. 1) Most of the studies were conducted in Asia (46%, 26 of 56), followed by the Americas (20%, 11 of 56), and Europe (20%, 11 of 56). The characteristics of each study are shown in Additional file 1: Table S2 [25–80].

### Risk factors for MASLD among children and adolescents

We synthesized odds ratios for seven risk factors, including four factors in the main analysis and three additional factors treated as exploratory. Among the main risk factors, BMI-Z score (OR 1.81 [95% CI: 1.14, 2.89]) and male sex (OR 1.77 [1.50, 2.10]) were significantly associated with MASLD among 5–24-year-olds. BMI showed a borderline association with MASLD (OR 1.12 [0.98, 1.27], *P* = 0.095), with considerable heterogeneity across studies (Table 1, Additional file 1: Table S3) Given the strength of available evidence and data availability, we further used BMI to analyze differences in MASLD prevalence across different populations. The age- and sex-specific ORs for overweight and obese individuals relative to other populations are provided in Additional file 1: Table S4.

**Table 1:** Synthesized effect size for risk factors for MASLD, reported for risk factors evaluated in at least five studies#

Risk factors	Number of studies	Number of participants	Odds ratio (95% CI)	*P* value
BMI	6	9212	1.12 (0.98, 1.27)	0.095
BMI-Z	5	5662	1.81 (1.14, 2.89)	0.013
Male	8	9669	1.77 (1.50, 2.10)	< .001
Age	5	4494	0.97 (0.78, 1.20)	0.777

*MASLD *metabolic dysfunction-associated steatotic liver disease, *BMI *body mass index, *IR *insulin resistance, *ALT *alanine aminotransferase, *TG *triglyceride. # See Table S3 for additional exploratory analysis

### Global and age-sex-specific prevalence of MASLD in 5–24-year-olds, 2000–2050

**Fig. 2:**
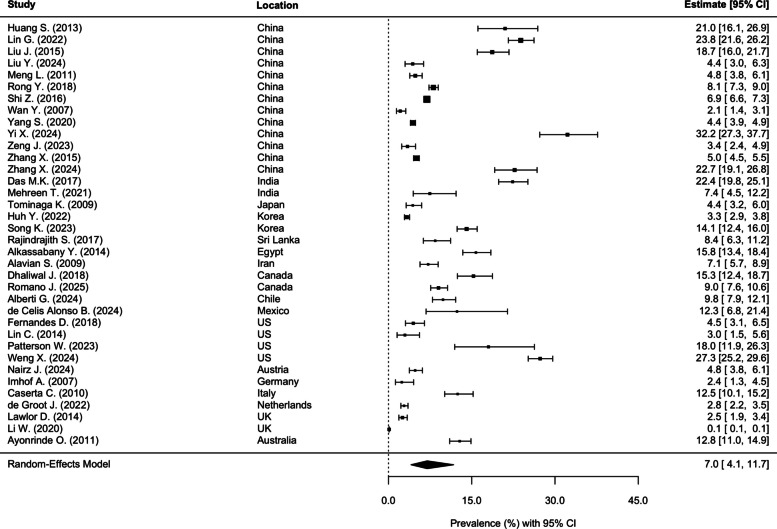
Pooled prevalence of global MASLD among children and adolescents aged 5 to 24 years. Note: MASLD, metabolic dysfunction-associated steatotic liver disease; CI, confidence interval

Globally, the prevalence of MASLD among 5–24-year-olds was 7.0% (95% CI: 4.1, 11.7) (Fig. 2). Substantial between-study heterogeneity (*I*^2^ = 98.2%) was observed in the multilevel random-effects meta-analysis. Influence diagnostics and leave-one-out analyses showed that omitting any single study did not materially change the pooled prevalence estimate, indicating that the results were not driven by individual studies. Assessment of small-study effects revealed no evidence of funnel plot asymmetry, with Egger’s regression test being non-significant (*p* = 0.436). (Additional file 1: Fig. S1).

In general, the prevalence of MASLD increased with increasing age and investigation year (recency), with boys experiencing a higher prevalence than girls. For example, the prevalence of MASLD among girls and boys aged 5 to 9 years increased from 1.8% (0.7, 4.3) and 4.4% (1.8, 10.2) in 2000 to 5.0% (2.1, 11.4) and 11.8% (5.2, 24.7) in 2020, and is predicted to increase to 5.7% (0.2, 10.9) and 13.4% (2.4, 24.4) in 2050. This prevalence further increased with age, reaching 16.2% (1.3, 31.2) and 33.4% (9.1, 57.5) in 20–24-year-old females and males, respectively, in 2050 (Table 2). The sensitivity analysis yielded higher prevalence values, while the overall pattern of results remained similar (Additional file 1: Table S5).

**Table 2:** Estimated (2000, 2010, 2020) and predicted (2030, 2040, 2050) global age-sex-specific prevalence of MASLD in children and adolescents aged 5 to 24 years

Age	2000		2010		2020		2030		2040		2050	
	Girls	Boys	Girls	Boys	Girls	Boys	Girls	Boys	Girls	Boys	Girls	Boys
5–9	1.8 (0.7, 4.3)	4.4 (1.8, 10.2)	3.0 (1.3, 6.5)	7.3 (3.4, 15.1)	5.0 (2.1, 11.4)	11.8 (5.2, 24.7)	5.2 (0.4, 10.0)	12.2 (2.1, 22.5)	5.4 (0.2, 10.4)	12.8 (2.1, 23.5)	5.7 (0.2, 10.9)	13.4 (2.4, 24.4)
10–14	2.4 (1.2, 4.9)	6.0 (3.0, 11.5)	4.1 (2.3, 7.1)	9.7 (5.6, 16.4)	6.7 (3.5, 12.6)	15.5 (8.4, 26.9)	7.3 (2.4, 12.2)	16.7 (6.8, 26.8)	7.9 (2.5, 13.3)	18.1 (7.4, 28.9)	8.6 (2.7, 14.5)	19.8 (8.2, 31.6)
15–19	3.3 (1.5, 6.9)	8.0 (3.9, 15.9)	5.5 (2.9, 10.1)	12.9 (7.1, 22.2)	9.0 (4.5, 17.1)	20.1 (10.7, 34.5)	9.8 (3.0, 16.6)	21.5 (8.8, 34.1)	10.5 (3.1, 17.9)	23.0 (9.3, 36.3)	11.4 (3.5, 19.4)	24.8 (10.2, 39.2)
20–24	4.5 (1.6, 11.6)	10.7 (4.1, 25.2)	7.4 (3.0, 16.9)	16.9 (7.4, 34.2)	11.9 (4.8, 26.7)	25.7 (11.4, 48.2)	13.4 (1.3, 25.3)	28.0 (8.4, 47.8)	15.0 (1.5, 28.6)	30.8 (8.7, 53.3)	16.2 (1.3, 31.2)	33.4 (9.1, 57.5)

*MASLD *metabolic dysfunction-associated steatotic liver disease

### Global and regional trends in the number of cases of MASLD, 2000–2050

**Fig. 3:**
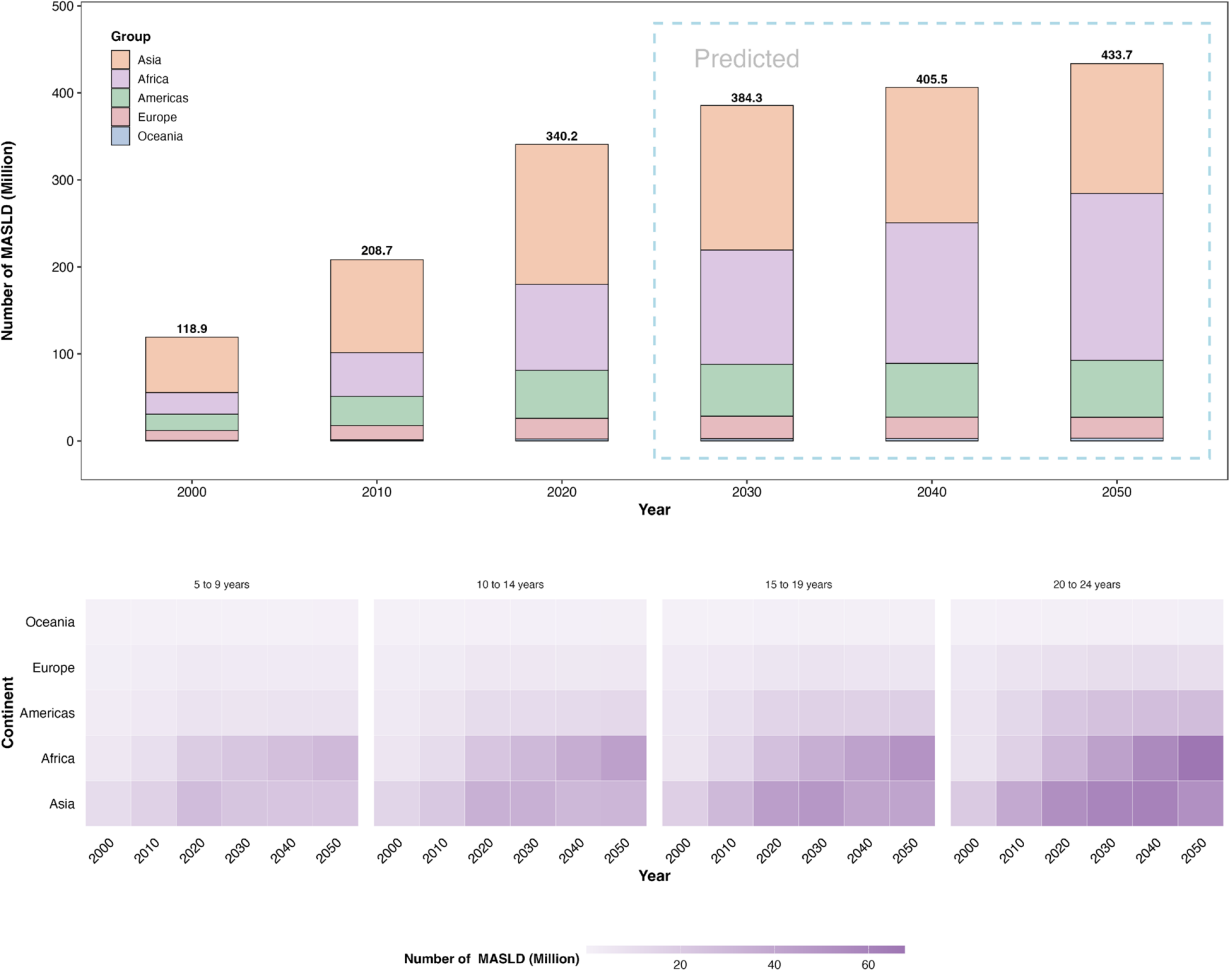
Regional estimated (2000, 2010, 2020) and predicted (2030, 2040, 2050) numbers of children and adolescents with MASLD. Note: MASLD, metabolic dysfunction-associated steatotic liver disease

Globally, the number of cases of MASLD among 5–24-year-olds increased from 118.9 million (95% CI: 52.2, 260.5) in 2000 to 340.2 million (162.8, 650.3) in 2020. This is predicted to further increase to 433.7 million (122.8, 744.0) by 2050. Regionally, Asia, Africa and the Americas were the three regions with the highest number of MASLD cases during 2000 and 2020, where the number of cases increased from 63.8 million (51.5, 76.6), 24.9 million (20.3, 29.4), and 18.8 million (15.4, 22.2) in 2000 to 160.9 million (134.5, 187.6), 98.9 million (84.7, 113.1), and 55.2 million (47.2, 63.3) in 2020, respectively. Under the modeled framework, Africa is projected to surpass Asia from 2040 onward to become the region with the highest number of cases. In 2050, the number of cases of MASLD is predicted to increase to 149.2 million (123.6, 175.4), 191.9 million (163.4, 219.6), and 65.4 million (55.3, 75.4) in Asia, Africa, and Americas, respectively (Fig. 3).

### National and provincial trends of MASLD prevalence among Chinese children and adolescents, 2000–2050

**Fig. 4:**
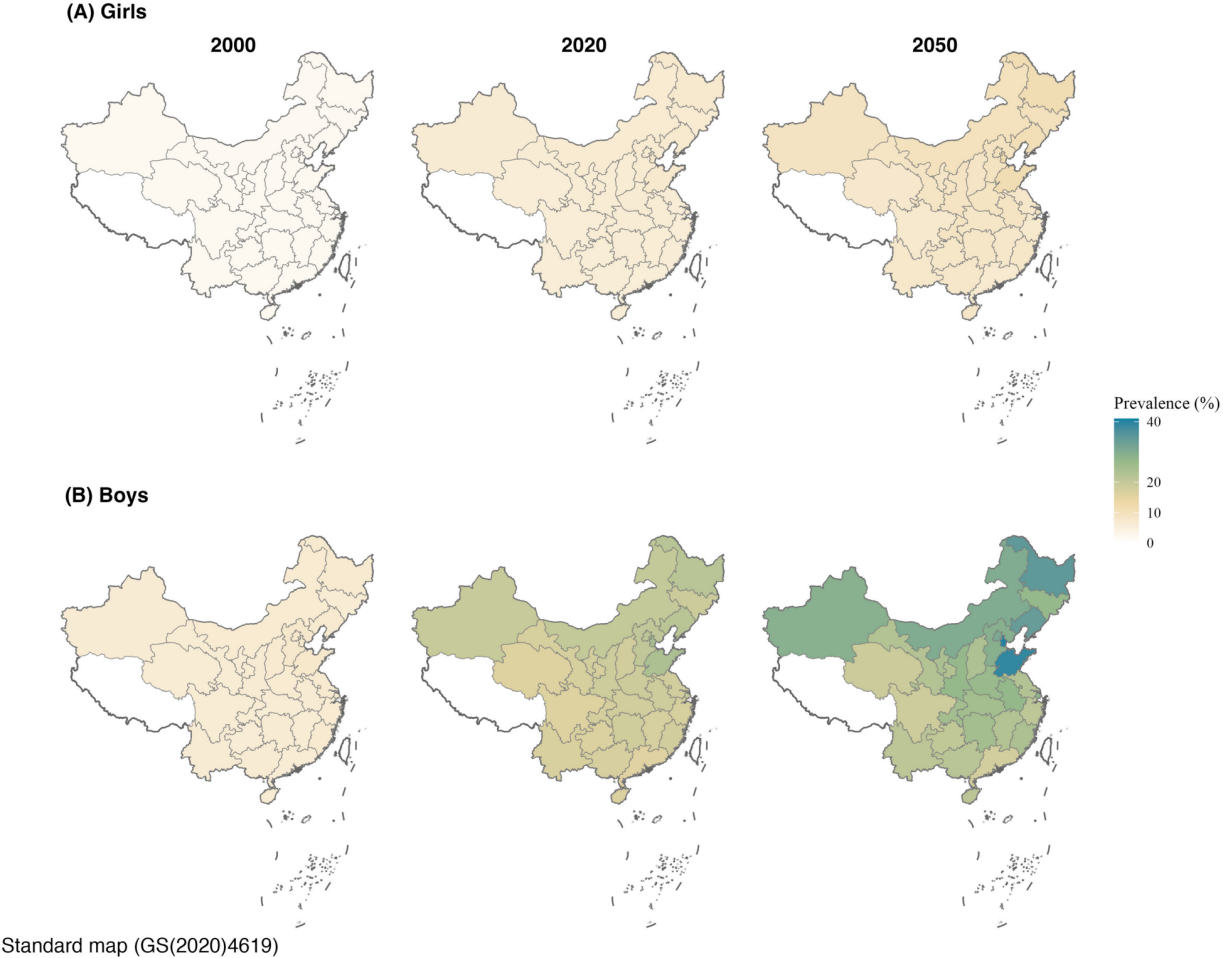
Prevalence of MASLD among children and adolescents aged 6 to 18 years in China. **A** Girls. **B** Boys. Note: MASLD, metabolic dysfunction-associated steatotic liver disease

The national number of cases of MASLD among Chinese 5–24-year-olds increased from 23.5 million (95% CI: 15.1, 32.1) in 2000 to 51.5 million (37.5, 65.3) in 2020. Predictions suggest cases will peak at 54.5 million (39.1, 69.5) in 2030, and then decline to 36.0 million (25.0, 47.0) by 2050 (Additional file 1: Table S6). At the provincial level in 6–18-year-olds, the highest prevalence in 2000 was observed in Hebei (MASLD prevalence was 2.4% [1.2, 3.5] and 7.8% [4.4, 11.3] for girls and boys, respectively), Shandong (2.3% [1.2, 3.4] for girls, 7.8% [4.4, 11.2] for boys), and Beijing (2.3% [1.2, 3.4] for girls, 7.5% [4.2, 10.8] for boys). In 2020, the highest prevalence was observed in Tianjin (7.9% [4.8, 11.0] for girls, 24.2% [16.3, 32.1] for boys), Shandong (7.6% [4.7, 10.5] for girls, 23.8% [16.1, 31.6] for boys), and Heilongjiang (7.4% [4.5, 10.3] for girls, 21.9% [14.6, 29.1] for boys). Under the modeled framework, the top three provinces are predicted to remain Tianjin (12.4% [6.7, 18.1] for girls, 40.1% [23.5, 56.8] for boys), Shandong (11.7% [6.4, 16.9] for girls, 39.3% [23.2, 55.4] for boys), and Heilongjiang (11.5% [6.2, 16.8] for girls, 34.8% [20.1, 49.5] for boys) by 2050 (Fig. 4, Additional file 1: Fig. S2, Table S7).

## Discussion

In this study we found that globally, 7.0% of children and adolescents aged 5 to 24 years experienced MASLD. The prevalence varied by age group, sex, year, and region. Under the modeled framework, the estimated prevalence was highest in 20–24-year-old males, with cases increasing from 118.9 million in 2000 to 340.2 million in 2020 and projected to reach 405.5 million by 2050. Our model indicated that the populations affected by MASLD were primarily concentrated in Asia and Africa, with a gradual shift towards Africa predicted over time. In China, the total affected population among 5–24-year-olds was estimated to increase from 23.5 million in 2000 to 51.5 million in 2020, to peak at 54.5 million in 2030, and to subsequently decline to 36.0 million by 2050. At the provincial level, among Chinese girls and boys aged 6 to 18 years, prevalence was estimated to be highest in northern provinces such as Tianjin, Shandong, and Heilongjiang, with an upward trend in all provinces over time.

Our pooled global prevalence (7.0%) was lower than that reported by Anderson et al. in 2015 (7.6%), Li et al. in 2022 (7.4%), and especially by Lee et al. in 2024 (13%) [4, 81, 82]. This discrepancy may partly be attributable to differences in the databases searched and the inclusion and exclusion criteria, which resulted in us incorporating more studies [37] than in these three reports (20, 14, and 27 studies, respectively). Additional minor differences may also arise from the number of papers reporting on the same population; in this study, we sought to include only the largest and most representative paper from each population. MASLD prevalence was consistently higher in boys than in girls, a pattern that aligns with prior global and regional evidence in children and adolescent populations [4, 82]. This sex disparity has been partly attributed to higher levels of insulin resistance and greater visceral adiposity in boys [83].

While a clinical diagnosis of MASLD is often made between 10 and 13 years of age [84], this does not imply that children develop the condition at this age. MASLD may well be present earlier [84], and in this study, the literature indeed reports cases in younger age groups [44]. We currently have limited understanding of the impact of early and long-term exposure to MASLD in pediatric populations. Previous studies have shown that obesity during young adulthood is an important risk factor for MASLD in middle and later adulthood [85], and that exposure to MASLD during childhood significantly increases premature mortality and hepatic as well as extrahepatic comorbidities over an average follow-up of 8.5 years [5]. Whether such early and long-term exposure of MASLD confers additional adverse effects in adulthood and later life remains to be further investigated. Our modeling indicates that the prevalence of MASLD increases with age. Given the close association between MASLD and other metabolic syndromes, as well as its characteristic clustering with multiple risk factors, this age-related increase underscores the importance of initiating comprehensive preventive and management strategies early in the life course in an effort to mitigate the subsequent disease burden [86].

A few studies have reported trends in the burden of MASLD in children and adolescents [87–90], most of which used the GBD study [87–89]. The reliability of GBD estimates is largely contingent upon both the quantity and quality of the underlying data sources. A central feature of GBD methodology is the application of the ST-GPR framework, which enables borrowing strength from adjacent time points, age groups and location to achieve smoothing across age, time, and location, particularly in settings with incomplete datasets [14]. Generally considered a strength, this smoothing process may introduce bias when the data borrowed from neighboring locations are themselves of limited reliability. For instance, in GBD 2021, when examining children and adolescents in China, the estimated prevalence of MASLD demonstrated only minor fluctuations with a slight upward trend [13], a pattern that appears inconsistent with China’s rapidly rising obesity rates. In contrast, our study was based on a meta-analysis of MASLD and employed modeling grounded in more extensively reported and data-rich estimates of overweight and obesity, which we believe provides a more robust capacity to capture the underlying trends. A number of studies have utilized systematic review and meta-analytic approaches to assess the prevalence and predict the trends of NAFLD among children and adolescents [81, 91, 92]. Nevertheless, these studies failed to develop comprehensive models and did not provide an in-depth analysis of the heterogeneity in prevalence across subgroups or among populations with varying OWOB prevalence. Another study based on NHANES and NCD risk data estimated the prevalence of MASLD using BMI and projected its burden in 2030 [90]. However, our methodological approach differs fundamentally. While their inputs relied on NHANES data, our modeling incorporated epidemiological data from multiple countries worldwide. By leveraging multinational meta-analytic data and integrating projected obesity prevalence, we further estimated the burden of MASLD through to 2050, additionally accounting for the influence of population size. While model-based estimates suggest that MASLD cases were primarily concentrated in Asia and Africa, with a relatively rapid shift toward Africa, this pattern should be interpreted cautiously given the sparsity of primary epidemiological data on MASLD in African population and the greater reliance on modeled risk-factor and demographic assumptions. In these regions, the prevalence of overweight and obesity has been continuously increasing and is projected to further increase between 2030 and 2050 [16]; accordingly, the prevalence of MASLD is also projected to increase. The declining population of children and adolescents in Asia (from 1336 million in 2000 to 1062 million in 2050), together with the concurrent projected population growth in Africa (from 501 million in 2000 to 1118 million in 2050), is likely to explain the rapid shift of MASLD towards Africa. Most African countries are of low- and middle-income, where there are important opportunities to reduce overweight and obesity among children and adolescents through a range of measures such as improving food systems, supporting antenatal care and breastfeeding, implementing school nutrition programs, and promoting health education [16]. Achieving these goals, which will also lessen the population burden of MASLD, will require effective cross-sectoral coordination and collaboration [93].

Within Asia, China serves as an illustrative case, having undergone rapid economic development and profound shifts in nutritional status over recent decades [94], while also displaying striking heterogeneity across its provinces. Modeled-based estimates suggest that the total number of cases of MASLD will peak in 2030 and then gradually decline, even though the prevalence in China is expected to continue rising in parallel with increasing trends in overweight and obesity. At the provincial level, model-based estimates indicate that persistently high MASLD rates will be concentrated in the more economically developed regions of northern China; however, some less developed provinces, such as Heilongjiang, have also ranked among the third highest in nationwide prevalence since 2020. This spatial pattern is consistent with prior evidence, which reported that MASLD prevalence among pediatric populations in northern China was consistently higher than in southern regions [95]. Previous studies have also noted that the northern provinces were hot spots of overweight and obesity among children and adolescents in China [96], which is consistent with the rapid increases in MASLD prevalence observed in these less developed northern areas. This may be attributable to rapid economic development and related social determinants [96, 97], which has enabled children and adolescents across different regions to more easily access energy-dense foods, often accompanied by insufficient physical activity. In relatively less developed areas, however, health education and supporting facilities have progressed more slowly [98], making it difficult to cope with the rapidly increasing rates of overweight and obesity as well as MASLD. These areas may additionally experience a dual burden of undernutrition and overnutrition [99, 100], which further complicates the implementation of effective interventions. Urban–rural differences were not explicitly incorporated into our modeling framework, and the available data were predominantly derived from urban populations, which may limit the generalizability of the model to rural settings and reflects persistent gaps in equity and surveillance. Under the modeled framework, the transitional patterns observed in China resemble the global shift of focus toward Africa, underscoring the need to strengthen health investments and education in less developed areas, promote healthy diets and physical activity in all regions, and ensure that profit-driven commercial determinants are tempered by health concerns [93].

Our study has several limitations. First, substantial heterogeneity across studies may limit comparability, reflecting differences in diagnostic definitions, assessment methods, and study contexts. Sensitivity analyses restricted to imaging- or biopsy-based diagnoses yielded broadly consistent results, supporting the robustness of our findings despite persistent heterogeneity. Second, although MASLD was used as the outcome term throughout this study, our inclusion of studies adopting different diagnostic frameworks (NAFLD, MAFLD, and MASLD) may have introduced some degree of definitional heterogeneity [101]. These diagnostic frameworks are closely related and share a common phenotype of hepatic steatosis, and prior studies have shown substantial overlap between NAFLD and MASLD populations [18]. Nevertheless, subtle differences in diagnostic criteria across studies cannot be entirely excluded and may have contributed to uncertainty in prevalence estimates. Third, because our analysis was based on cross-sectional data, it could not capture the temporal aspects of MASLD development, specifically, the duration of OWOB or the disease’s progression or staging, which should be further explored in future research. Fourth, our projections rely on several modeling assumptions, including the transferability of odds ratios for overweight and obesity across populations and over time, simplified additive effects at the population level, and assumptions regarding future obesity and demographic trends. These factors may not fully capture nonlinearity or effect modification, and projections should therefore be interpreted as scenario-based rather than deterministic estimates. Fifth, we integrated data from multiple sources, which inevitably introduces the inherent limitations of each dataset. Nevertheless, all data employed were published, well-documented and broadly validated [16, 21–23]. Sixth, due to data availability and our aim to include as many time points as possible for better predictions, our subnational analysis in China included only Han students aged 6–18 years. Extrapolation of our conclusions to other ethnic groups or those no longer attending school should be done with caution. In future, more comprehensive data will be needed for further validation and investigation.

## Conclusions

In summary, our model-based analyses suggest that, despite sex- and age-specific differences, the prevalence of MASLD among children and adolescents increased up to 2020, and may continue to rise through 2050 in parallel with the growing obesity epidemic. Globally, our model indicates a potential shift in the burden of MASLD toward Africa, largely driven by demographic change. Our findings in China also reveal a growing risk of MASLD in currently less developed regions under the modeled framework. Effective cross-sectoral coordination and collaboration coupled with holistic healthcare initiatives and increased investment in healthcare and health education are crucial in less developed regions to curb the rise of MASLD and to improve health outcomes in childhood and beyond.

## Supplementary Information

Additional file 1. Supplementary Figures and Tables. Table S1. Search strategy to identify studies reporting the prevalence of MASLD. Table S2. Characteristic of different studies. Table S3. Exploratory associations between selected risk factors and MASLD explored in less than five studies. Table S4. Age-sex-specific ORs of MASLD for overweight and obese (OWOB) population relative to non-OWOB population. Table S5. Estimated (2000, 2010, 2020) and predicted (2030, 2040, 2050) global age-sex-specific prevalence of MASLD in children and adolescents aged 5 to 24 years, in five-year age bands. Table S6. National number of MASLD among Chinese children and adolescents aged 5 to 24 years, 2000~2050. Table S7. Provincial prevalence of MASLD among Chinese children and adolescents aged 6 to 18 years, 2000~2050. Fig. S1. Leave-one-out analysis (A) and funnel plot (B). Fig. S2. Increase in MASLD prevalence among children and adolescents aged 6–18 years in China from 2000 to 2050 (A): Girls; (B): Boys.

Additional file 2.

## Data Availability

The data used for meta-analysis could be found in the Additional file 1: Table S2. The GBD and World Population Prospects data could be found in [https://vizhub.healthdata.org/gbd-results/] and [https://population.un.org/wpp/], respectively. The Analytic code and CNSSCH data are available from the authors upon reasonable request.

## Abbreviations

ALT: Alanine aminotransferase
BMI: Body mass index
CI: Confidence interval
CNKI: China National Knowledge Infrastructure
CNSSCH: Chinese National Survey on Students’ Constitution and Health
DALYs: Disability-adjusted life-years
GBD: Global Burden of Disease
HALE: Healthy life expectancy
IOTF: International Obesity Task Force
IR: Insulin resistance
MAFLD: Metabolic dysfunction-associated fatty liver disease
MASLD: Metabolic dysfunction-associated steatotic liver disease
NAFLD: Nonalcoholic fatty liver disease
OR: Odds ratio
OWOB: Overweight or obese
ST-GPR: Spatiotemporal Gaussian Process Regression
STROBE: Strengthening the Reporting of Observational Studies in Epidemiology
TG: Triglycerides
