# Supplementary material for: Global and regional prevalence, burden, and risk factors for MASLD in children and adolescents aged 5 to 24 years: a systematic review, meta-analysis, and modeling study
Source: BMC Med. 2026 Mar 18;24:267. doi: 10.1186/s12916-026-04801-3 (PMC13113483; doi:10.1186/s12916-026-04801-3)
Supplement: Supplementary file 1 — Additional file 1. Supplementary Figures and Tables. Table S1. Search strategy to identify studies reporting the prevalence of MASLD. Table S2. Characteristic of different studies. Table S3. Exploratory associations between selected risk factors and MASLD explored in less than five studies. Table S4. Age-sex-specific ORs of MASLD for overweight and obese (OWOB) population relative to non-OWOB population. Table S5. Estimated (2000, 2010, 2020) and predicted (2030, 2040, 2050) global age-sex-specific prevalence of MASLD in children and adolescents aged 5 to 24 years, in five-year age bands. Table S6. National number of MASLD among Chinese children and adolescents aged 5 to 24 years, 2000~2050. Table S7. Provincial prevalence of MASLD among Chinese children and adolescents aged 6 to 18 years, 2000~2050. Fig. S1. Leave-one-out analysis (A) and funnel plot (B). Fig. S2. Increase in MASLD prevalence among children and adolescents aged 6–18 years in China from 2000 to 2050 (A): Girls; (B): Boys. [file 12916_2026_4801_MOESM1_ESM.docx]

Table S1. Search strategy to identify studies reporting the prevalence of MASLD

| Database | Search terms |
| --- | --- |
| PubMed  （732） | (MASLD[Title/Abstract] OR NAFLD[Title/Abstract] OR MAFLD[Title/Abstract] OR metabolic dysfunction-associated steatotic liver disease[Title/Abstract] OR metabolic dysfunction-associated fatty liver disease[Title/Abstract] OR nonalcoholic fatty liver disease[Title/Abstract]) AND  (prevalence[Title/Abstract] OR epidemiology[Title/Abstract]) AND (children[Title/Abstract] OR adolescents[Title/Abstract] OR child[Title/Abstract] OR adolescent[Title/Abstract] OR teenager[Title/Abstract]) |
| EMBASE (1980-)  （1270） | (MASLD OR NAFLD OR MAFLD OR 'metabolic dysfunction-associated steatotic liver disease' OR 'metabolic dysfunction-associated fatty liver disease' OR 'nonalcoholic fatty liver disease'):ab,ti AND ((prevalence OR epidemiology):ab,ti) AND ((children OR adolescents OR child OR adolescent OR teenager):ab,ti) |
| Web of Science  (636) | (MASLD OR NAFLD OR MAFLD OR 'metabolic dysfunction-associated steatotic liver disease' OR 'metabolic dysfunction-associated fatty liver disease' OR 'nonalcoholic fatty liver disease'):ab AND ((prevalence OR epidemiology):ab) AND ((children OR adolescents OR child OR adolescent OR teenager):ab) |
| Cochrane  （82） | (MASLD OR NAFLD OR MAFLD OR 'metabolic dysfunction-associated steatotic liver disease' OR 'metabolic dysfunction-associated fatty liver disease' OR 'nonalcoholic fatty liver disease'):ab,ti AND ((prevalence OR epidemiology):ab,ti) AND ((children OR adolescents OR child OR adolescent OR teenager):ab,ti) |
| CNKI  (27) | (TI % '脂肪肝') AND (SU % '患病率'+ '罹患率' + '现患率'+ '流行' + '调查'+ '现况') AND (SU % '儿童'+ '青少年' + '学生') |

Note: CNKI= China National Knowledge Infrastructure.

Table S2 Characteristic of different studies

| First author | Year published | Country | GBD region | Continent | Population | Diagnose | Prevalence synthetizes | MV analyses on risk factors | Sample size | Age (mean/median, years) | Female proportion (%) | Selection (sample population) risk score | Selection (sample size) risk score | Selection (participation rate) risk score | Performance bias (outcome assessment) score | Performance bias (analytical methods to control for bias) score |
| --- | --- | --- | --- | --- | --- | --- | --- | --- | --- | --- | --- | --- | --- | --- | --- | --- |
| Alavian S. | 2009 | Iran | North Africa and Middle East | Africa | Normal | Ultrasound | Yes | Yes | 966 | 12.5 | 55.2 | 2 | 0 | 0 | 2 | 2 |
| Alkassabany Y. | 2014 | Egypt | North Africa and Middle East | Africa | Normal | Ultrasound | Yes | Yes | 800 | 12.0 | 56.4 | 0 | 0 | 2 | 2 | 2 |
| Dehnavi Z. | 2021 | Iran | North Africa and Middle East | Africa | OWOB | CAP | No | Yes | 70 | 12.8 | 52.8 | 0 | 2 | 0 | 2 | 2 |
| Imanzadeh F. | 2023 | Iran | North Africa and Middle East | Africa | OB | Ultrasound & ALT | No | Yes | 115 | 12.6 | 38.0 | 2 | 1 | 0 | 2 | 2 |
| Mburu A. | 2023 | Kenya | Eastern Sub-Saharan Africa | Africa | OWOB | Ultrasound | No | Yes | 103 | 10.0 | 57.3 | 0 | 1 | 0 | 1 | 1 |
| Namakin K. | 2018 | Iran | North Africa and Middle East | Africa | OB | Ultrasound | No | Yes | 200 | 15.0 | 57.0 | 1 | 1 | 2 | 2 | 2 |
| Özer Y. | 2024 | Turkey | North Africa and Middle East | Africa | OB | Ultrasound | No | Yes | 155 | 13.8 | 72.0 | 0 | 0 | 0 | 2 | 1 |
| Alberti G. | 2024 | Chile | Southern Latin America | Americas | Normal | Ultrasound | Yes | Yes | 784 | 15.4 | 51.5 | 1 | 2 | 0 | 2 | 2 |
| Dhaliwal J. | 2018 | Canada | High-income North America | Americas | Normal | CT | Yes | No | 503 | 9.5 | 49 | 1 | 1 | 0 | 2 | 2 |
| Damaso A. | 2008 | Brazil | Tropical Latin America | Americas | OB | Ultrasound | No | Yes | 68 | 16.69 | 62 | 0 | 0 | 0 | 2 | 2 |
| Fernandes D. | 2018 | United States of America | High-income North America | Americas | Normal | Biopsy | Yes | Yes | 582 | 15.1 | 23 | 1 | 1 | 0 | 2 | 2 |
| Felix D. | 2016 | Brazil | Tropical Latin America | Americas | OB | Ultrasound | N0 | Yes | 39 | 8.8 | 56.4 | 0 | 1 | 0 | 2 | 2 |
| Lin C. | 2014 | United States of America | High-income North America | Americas | Normal | Ultrasound | Yes | Yes | 304 | 7.7 | 49 | 1 | 0 | 0 | 1 | 1 |
| Patterson W. | 2023 | United States of America | High-income North America | Americas | Normal | MRI | Yes | Yes | 111 | 19.5 | 45 | 1 | 0 | 1 | 2 | 2 |
| Romano J. | 2025 | Canada | High-income North America | Americas | Normal | NRS | Yes | No | 1365 | 13 | 49.0 | 2 | 2 | 0 | 1 | 2 |
| Weng X. | 2024 | United States of America | High-income North America | Americas | Normal | CAP | Yes | Yes | 1559 | 15 | 47.3 | 2 | 1 | 2 | 2 | 1 |
| de Celis Alonso B. | 2024 | Mexico | Central Latin America | Americas | Normal | MRI | Yes | No | 81 | 8.2 | 0 | 0 | 2 | 2 | 2 | 2 |
| do Nascimento J. | 2013 | Brazil | Tropical Latin America | Americas | Normal & OB | MRI | No | Yes | 50 | 14.4 | 50 | 0 | 0 | 0 | 2 | 1 |
| Chen X. | 2021 | China | East Asia | Asia | OB | Ultrasound | No | Yes | 286 | 10.38 | 46.9 | 0 | 1 | 1 | 2 | 2 |
| Das M. | 2017 | India | South Asia | Asia | Normal | Ultrasound | Yes | No | 961 | 7.5 | 45 | 2 | 1 | 0 | 2 | 2 |
| Duan Y. | 2022 | China | East Asia | Asia | OB | Ultrasound | No | Yes | 267 | 12 | 16 | 0 | 0 | 0 | 2 | 2 |
| Gupta N. | 2020 | India | South Asia | Asia | OB | Ultrasound | No | Yes | 100 | 10.6 | 46 | 0 | 2 | 0 | 2 | 2 |
| Huang S. | 2013 | China | East Asia | Asia | Normal | Ultrasound | Yes | No | 219 | 9 | 36.1 | 2 | 1 | 0 | 2 | 0 |
| Huh Y. | 2022 | Republic of Korea | High-income Asia Pacific | Asia | Normal | ALT | Yes | No | 5685 | 15 | 47 | 2 | 1 | 1 | 2 | 2 |
| Jin B. | 2024 | China | East Asia | Asia | OB | Ultrasound | No | Yes | 2081 | 10.61 | 30.6 | 1 | 0 | 2 | 2 | 2 |
| Lin G. | 2022 | China | East Asia | Asia | Normal | CAP | Yes | Yes | 1301 | 13.31 | 73.5 | 2 | 1 | 2 | 1 | 1 |
| Liu J. | 2015 | China | East Asia | Asia | Normal | Ultrasound | Yes | No | 717 | 20.5 | 45 | 2 | 0 | 1 | 2 | 2 |
| Liu Y. | 2024 | China | East Asia | Asia | Normal | Ultrasound & CAP | Yes | No | 1018 | 10 | 44 | 2 | 1 | 0 | 1 | 1 |
| Mehreen T. | 2021 | India | South Asia | Asia | Normal | Ultrasound | Yes | No | 188 | 20 | 51 | 0 | 1 | 2 | 2 | 1 |
| Meng L. | 2011 | China | East Asia | Asia | Normal | Ultrasound | Yes | Yes | 1449 | 12 | 44.2 | 1 | 0 | 2 | 2 | 1 |
| Pawar S. | 2016 | India | South Asia | Asia | OWOB | Ultrasound & ALT | No | Yes | 100 | 13 | NA | 1 | 1 | 2 | 2 | 2 |
| Rajindrajith S. | 2017 | Sri Lanka | Southeast Asia | Asia | Normal | Ultrasound | Yes | No | 499 | 14 | 51.5 | 2 | 2 | 0 | 1 | 2 |
| Rong Y. | 2018 | China | East Asia | Asia | Normal | Ultrasound | Yes | No | 4141 | 18.62 | 49.8 | 2 | 2 | 2 | 2 | 2 |
| Shi Z. | 2016 | China | East Asia | Asia | Normal | ALT & OW | Yes | No | 19162 | 18.1 | 49.4 | 2 | 2 | 2 | 2 | 2 |
| Song K. | 2023 | Republic of Korea | High-income Asia Pacific | Asia | Normal | ALT | Yes | Yes | 1428 | 14 | 47.4 | 2 | 1 | 2 | 2 | 2 |
| Tominaga K. | 2009 | Japan | High-income Asia Pacific | Asia | Normal | Ultrasound | Yes | No | 846 | 10.5 | 48.2 | 2 | 1 | 1 | 2 | 2 |
| Wan Y. | 2007 | China | East Asia | Asia | Normal | Ultrasound | Yes | No | 1180 | 9 | 51.5 | 1 | 1 | 2 | 2 | 2 |
| Yang S. | 2020 | China | East Asia | Asia | Normal | Ultrasound | Yes | No | 7759 | 9.09 | 22.6 | 1 | 1 | 2 | 2 | 2 |
| Yi X. | 2024 | China | East Asia | Asia | Normal | Ultrasound_ | Yes | Yes | 307 | 12.2 | 49.9 | 2 | 1 | 0 | 2 | 2 |
| Zeng J. | 2023 | China | East Asia | Asia | Normal | CAP | Yes | No | 848 | 8 | 49.2 | 2 | 1 | 0 | 2 | 2 |
| Zhang X. | 2015 | China | East Asia | Asia | Normal | Ultrasound | Yes | Yes | 7229 | 12.3 | 49 | 2 | 1 | 2 | 2 | 2 |
| Zhang X. | 2024 | China | East Asia | Asia | Normal | CAP | Yes | No | 462 | 9.92 | 53.9 | 0 | 1 | 2 | 2 | 2 |
| Zhou X. | 2022 | China | East Asia | Asia | OB | MRI | No | Yes | 3216 | 10.18 | 30.8 | 0 | 1 | 1 | 2 | 2 |
| de Silva M. | 2022 | Sri Lanka | Southeast Asia | Asia | OB | Ultrasound | No | Yes | 95 | 10.66 | 41 | 0 | 0 | 0 | 2 | 2 |
| Belei O. | 2017 | Romania | Central Europe | Europe | Normal | Ultrasound | No | Yes | 245 | 14 | 35.1 | 0 | 0 | 0 | 2 | 0 |
| Caserta C. | 2010 | Italy | Western Europe | Europe | Normal | Ultrasound | Yes | No | 642 | 12 | 50.6 | 2 | 1 | 1 | 2 | 0 |
| Denzer C. | 2009 | Germany | Western Europe | Europe | OB | Ultrasound | No | Yes | 241 | 13.5 | 55 | 0 | 0 | 2 | 2 | 2 |
| Di Sessa A. | 2021 | Italy | Western Europe | Europe | OB | Ultrasound | No | Yes | 318 | 12.31 | 100 | 0 | 0 | 0 | 2 | 2 |
| Imhof A. | 2007 | Germany | Western Europe | Europe | Normal | Ultrasound | Yes | No | 376 | 16 | 52 | 2 | 1 | 0 | 2 | 2 |
| Koot B. | 2011 | Netherlands | Western Europe | Europe | OB | Ultrasound | No | Yes | 144 | 14.1 | 63 | 0 | 1 | 1 | 1 | 0 |
| Lawlor D. | 2014 | United Kingdom | Western Europe | Europe | Normal | Ultrasound | Yes | Yes | 1711 | 17.9 | 57 | 2 | 0 | 2 | 2 | 2 |
| Li W. | 2020 | United Kingdom | Western Europe | Europe | Normal | Clinical record | Yes | No | 257746 | 17.5 | 52 | 1 | 0 | 2 | 1 | 2 |
| Nairz J. | 2024 | Austria | Western Europe | Europe | Normal | CAP | Yes | No | 1292 | 17.2 | 65.2 | 2 | 1 | 1 | 2 | 2 |
| Pacifico L. | 2020 | Italy | Western Europe | Europe | OWOB | Ultrasound | No | Yes | 234 | 12 | 44 | 0 | 0 | 0 | 2 | 2 |
| de Groot J. | 2022 | Netherlands | Western Europe | Europe | Normal | MRI | Yes | No | 2570 | 10.17 | NA | 2 | 2 | 0 | 2 | 0 |
| Ayonrinde O. | 2011 | Australia | Australasia | Oceania | Normal | Ultrasound | Yes | Yes | 1170 | 17 | 49 | 2 | 1 | 0 | 2 | 2 |

Table S3 Exploratory associations between selected risk factors and MASLD explored in less than five studies

| Risk factors | Number of studies | Number of participants | Odds ratio (95% CI) | P value |
| --- | --- | --- | --- | --- |
| Elevated ALT | 3 | 1176 | 4.88 (0.98, 24.36) | 0.053 |
| IR | 3 | 1334 | 2.79 (0.58, 13.43) | 0.200 |
| TG | 3 | 250 | 1.01 (1.00, 1.01) | 0.240 |

Note: MASLD, metabolic dysfunction-associated steatotic liver disease; BMI, Body Mass Index; IR, Insulin Resistance; ALT, Alanine Aminotransferase; TG, Triglyceride.

Table S4 Age-sex-specific ORs of MASLD for overweight and obese (OWOB) population relative to non-OWOB population

| Age | Girls | |  | Boys | |
| --- | --- | --- | --- | --- | --- |
|  | OW | OB |  | OW | OB |
| 5~9 | 1.56 | 2.38 |  | 1.52 | 2.24 |
| 10~14 | 1.98 | 4.19 |  | 1.88 | 3.71 |
| 15~19 | 2.08 | 4.41 |  | 1.98 | 3.96 |
| 20~24 | 2.08 | 4.37 |  | 2.00 | 3.97 |

Table S5 Estimated (2000, 2010, 2020) and predicted (2030, 2040, 2050) global age-sex-specific prevalence of MASLD in children and adolescents aged 5 to 24 years, in five-year age bands

| Age | 2000 | | 2010 | | 2020 | |  | 2030 | | 2040 | | 2050 | |
| --- | --- | --- | --- | --- | --- | --- | --- | --- | --- | --- | --- | --- | --- |
|  | Girls | Boys | Girls | Boys | Girls | Boys |  | Girls | Boys | Girls | Boys | Girls | Boys |
| 5~9 | 3.1 (1.3, 6.8) | 5.5 (2.4, 11.8) | 4.5 (2.6, 7.7) | 8.0 (4.7, 13.3) | 6.6 (3.2, 13.2) | 11.5 (5.7, 21.9) |  | 6.9 (1.5, 12.1) | 11.9 (3.6, 20.2) | 7.1 (1.6, 12.6) | 12.5 (3.9, 21.4) | 7.6 (1.8, 13.3) | 13.1 (3.7, 22.3) |
| 10~14 | 4.2 (2.1, 8.2) | 7.4 (3.8, 14.1) | 6.1 (4.4, 8.5) | 10.7 (7.8, 14.6) | 8.9 (5.0, 15.4) | 15.3 (8.9, 25.0) |  | 9.7 (4.0, 15.5) | 16.5 (7.9, 25.2) | 10.5 (4.4, 16.4) | 17.9 (8.5, 27.0) | 11.4 (4.7, 17.9) | 19.5 (9.2, 29.9) |
| 15~19 | 5.7 (2.7, 11.7) | 10.0 (4.8, 19.6) | 8.3 (5.4, 12.6) | 14.3 (9.4, 21.0) | 11.9 (6.4, 21.1) | 20.0 (11.2, 33.0) |  | 13.0 (5.1, 21.1) | 21.4 (9.8, 33.1) | 14.1 (5.5, 22.7) | 22.8 (10.4, 35.0) | 15.3 (5.8, 24.8) | 24.6 (10.9, 38.3) |
| 20~24 | 7.7 (2.9, 18.6) | 13.3 (5.3, 29.6) | 11.1 (5.4, 21.4) | 18.7 (9.5, 33.4) | 15.8 (7.0, 31.9) | 25.6 (12.1, 46.3) |  | 17.5 (3.8, 31.6) | 28.1 (9.5, 46.7) | 19.6 (4.0, 35.1) | 30.8 (10.7, 51.5) | 21.4 (4.5, 38.6) | 33.5 (11.4, 56.1) |

Note: Only studies utilizing ultrasound, FibroScan, computed tomography, magnetic resonance imaging, or liver biopsy were included in the analysis.

Table S6 National number of MASLD among Chinese children and adolescents aged 5 to 24 years, 2000~2050

| Year | Girls (million) | Boys (million) |
| --- | --- | --- |
| 2000 | 5.4 (2.8, 8.1) | 18.1 (10.1, 26.4) |
| 2010 | 9.0 (4.5, 13.6) | 29.7 (17.8, 41.7) |
| 2020 | 12.2 (7.0, 17.6) | 39.3 (26.5, 52.0) |
| 2030 | 13.1 (7.3, 19.2) | 41.3 (27.5, 55.3) |
| 2040 | 10.9 (5.0, 17.1) | 33.3 (19.5, 47.0) |
| 2050 | 9.1 (4.8, 13.7) | 26.9 (16.6, 37.1) |

Table S7 Provincial prevalence of MASLD among Chinese children and adolescents aged 6 to 18 years, 2000~2050

| Province | 2000 |  | 2010 |  | 2020 |  |  | 2030 |  | 2040 |  | 2050 |  |
| --- | --- | --- | --- | --- | --- | --- | --- | --- | --- | --- | --- | --- | --- |
|  | Girls | Boys | Girls | Boys | Girls | Boys |  | Girls | Boys | Girls | Boys | Girls | Boys |
| anhui | 2.1 (1.1, 3.1) | 6.6 (3.7, 9.5) | 3.7 (2.3, 5.0) | 11.1 (7.5, 14.7) | 6.5 (3.9, 9.0) | 18.8 (12.5, 25.0) |  | 7.1 (4.3, 10.0) | 21.0 (14.0, 28.0) | 7.9 (4.6, 11.2) | 24.1 (15.2, 32.9) | 9.0 (4.8, 13.2) | 27.2 (15.5, 39.0) |
| beijing | 2.3 (1.2, 3.4) | 7.5 (4.2, 10.8) | 4.2 (2.7, 5.6) | 12.9 (8.8, 17.0) | 7.1 (4.3, 9.8) | 21.3 (14.4, 28.3) |  | 7.9 (4.7, 11.0) | 23.8 (15.9, 31.6) | 8.7 (5.1, 12.3) | 27.1 (17.4, 36.8) | 10.0 (5.4, 14.6) | 30.9 (18.3, 43.6) |
| chongqing | 2.1 (1.1, 3.1) | 6.5 (3.6, 9.3) | 3.6 (2.3, 4.9) | 10.6 (7.2, 14.1) | 6.3 (3.8, 8.8) | 17.6 (11.9, 23.3) |  | 7.0 (4.2, 9.9) | 19.4 (12.9, 26.0) | 7.9 (4.6, 11.2) | 22.1 (14.0, 30.2) | 9.0 (4.8, 13.2) | 24.9 (14.2, 35.6) |
| fujian | 2.1 (1.1, 3.2) | 7.1 (4.0, 10.2) | 3.6 (2.3, 4.9) | 10.7 (7.2, 14.1) | 6.3 (3.8, 8.7) | 18.0 (12.0, 23.9) |  | 6.7 (4.0, 9.4) | 19.4 (12.7, 26.1) | 7.3 (4.3, 10.4) | 21.5 (13.4, 29.7) | 8.2 (4.3, 12.0) | 23.7 (13.1, 34.2) |
| gansu | 2.1 (1.1, 3.1) | 6.6 (3.7, 9.4) | 3.7 (2.4, 5.0) | 10.9 (7.5, 14.4) | 6.4 (3.9, 8.9) | 17.4 (11.7, 23.2) |  | 7.1 (4.2, 9.9) | 18.8 (12.6, 24.9) | 7.8 (4.6, 11.1) | 20.7 (13.2, 28.1) | 8.9 (4.8, 13.0) | 22.6 (13.2, 32.0) |
| guangdong | 2.1 (1.1, 3.1) | 6.3 (3.6, 9.1) | 3.6 (2.3, 4.9) | 10.7 (7.3, 14.2) | 5.9 (3.6, 8.1) | 15.8 (10.6, 20.9) |  | 6.2 (3.7, 8.7) | 16.3 (10.8, 21.7) | 6.6 (3.8, 9.3) | 17.2 (10.9, 23.4) | 7.1 (3.9, 10.4) | 17.9 (10.2, 25.5) |
| guangxi | 2.1 (1.1, 3.2) | 6.7 (3.8, 9.7) | 3.7 (2.4, 5.0) | 10.9 (7.4, 14.4) | 6.2 (3.8, 8.6) | 17.2 (11.6, 22.8) |  | 6.7 (4.0, 9.4) | 18.3 (12.2, 24.5) | 7.3 (4.3, 10.2) | 20.0 (12.7, 27.3) | 8.1 (4.4, 11.8) | 21.8 (12.6, 31.0) |
| guizhou | 2.1 (1.1, 3.1) | 6.3 (3.5, 9.1) | 3.7 (2.4, 5.0) | 10.7 (7.3, 14.0) | 6.2 (3.8, 8.6) | 16.6 (11.2, 22.0) |  | 6.7 (4.0, 9.4) | 17.5 (11.6, 23.4) | 7.3 (4.3, 10.3) | 19.0 (12.0, 26.0) | 8.2 (4.4, 12.0) | 20.6 (11.8, 29.3) |
| hainan | 2.1 (1.1, 3.1) | 6.5 (3.6, 9.4) | 3.6 (2.3, 4.8) | 10.5 (7.1, 13.8) | 6.2 (3.8, 8.6) | 16.6 (11.1, 22.2) |  | 6.8 (4.0, 9.5) | 17.8 (11.6, 24.1) | 7.5 (4.3, 10.7) | 19.7 (11.9, 27.4) | 8.4 (4.4, 12.5) | 21.5 (11.4, 31.6) |
| hebei | 2.4 (1.2, 3.5) | 7.8 (4.4, 11.3) | 3.9 (2.5, 5.3) | 12.5 (8.6, 16.4) | 6.9 (4.2, 9.6) | 20.6 (13.9, 27.3) |  | 7.6 (4.5, 10.7) | 22.8 (15.3, 30.3) | 8.5 (5.0, 11.9) | 25.9 (16.8, 35.0) | 9.6 (5.3, 13.9) | 29.3 (17.4, 41.2) |
| heilongjiang | 2.2 (1.2, 3.3) | 7.1 (4.0, 10.2) | 3.9 (2.5, 5.3) | 11.8 (8.0, 15.5) | 7.4 (4.5, 10.3) | 21.9 (14.6, 29.1) |  | 8.5 (5.1, 11.9) | 25.2 (16.7, 33.7) | 9.7 (5.7, 13.7) | 29.6 (18.7, 40.6) | 11.5 (6.2, 16.8) | 34.8 (20.1, 49.5) |
| henan | 2.2 (1.1, 3.2) | 6.8 (3.8, 9.8) | 3.9 (2.5, 5.3) | 12.0 (8.2, 15.8) | 6.7 (4.1, 9.2) | 19.0 (12.8, 25.1) |  | 7.4 (4.4, 10.3) | 20.9 (13.9, 27.8) | 8.2 (4.8, 11.5) | 23.5 (15.1, 31.8) | 9.3 (5.2, 13.5) | 26.4 (15.4, 37.4) |
| hubei | 2.1 (1.1, 3.1) | 6.4 (3.6, 9.2) | 3.7 (2.4, 5.0) | 11.1 (7.5, 14.6) | 6.3 (3.9, 8.8) | 18.2 (12.2, 24.1) |  | 6.9 (4.1, 9.7) | 19.8 (13.1, 26.5) | 7.6 (4.5, 10.8) | 22.2 (14.2, 30.2) | 8.6 (4.7, 12.5) | 25.0 (14.5, 35.5) |
| hunan | 2.1 (1.1, 3.2) | 6.7 (3.8, 9.6) | 3.7 (2.4, 5.0) | 11.5 (7.8, 15.1) | 6.3 (3.9, 8.7) | 18.2 (12.3, 24.2) |  | 6.8 (4.1, 9.6) | 19.9 (13.4, 26.5) | 7.5 (4.4, 10.5) | 22.3 (14.3, 30.2) | 8.4 (4.6, 12.2) | 24.8 (14.5, 35.1) |
| jiangsu | 2.2 (1.1, 3.2) | 7.1 (4.0, 10.2) | 3.8 (2.4, 5.1) | 11.9 (8.1, 15.6) | 6.3 (3.9, 8.7) | 18.0 (12.0, 24.0) |  | 6.7 (4.1, 9.4) | 19.1 (12.7, 25.5) | 7.2 (4.3, 10.2) | 20.9 (13.3, 28.4) | 8.0 (4.3, 11.6) | 22.6 (12.9, 32.2) |
| jiangxi | 2.1 (1.1, 3.1) | 6.5 (3.7, 9.3) | 3.6 (2.3, 4.9) | 10.6 (7.2, 14.1) | 6.2 (3.8, 8.6) | 17.3 (11.6, 23.0) |  | 6.7 (4.0, 9.3) | 18.6 (12.4, 24.9) | 7.2 (4.2, 10.3) | 20.5 (13.0, 27.9) | 8.1 (4.3, 11.9) | 22.5 (13.1, 31.9) |
| jilin | 2.2 (1.1, 3.2) | 6.5 (3.7, 9.4) | 3.8 (2.4, 5.1) | 11.4 (7.7, 15.0) | 6.9 (4.2, 9.5) | 18.8 (12.6, 25.1) |  | 7.7 (4.6, 10.9) | 21.0 (13.8, 28.2) | 8.7 (5.0, 12.3) | 23.9 (14.8, 33.0) | 10.0 (5.3, 14.8) | 27.0 (14.9, 39.2) |
| liaoning | 2.3 (1.2, 3.3) | 7.2 (4.0, 10.3) | 4.0 (2.6, 5.4) | 11.6 (7.9, 15.3) | 7.2 (4.4, 10.1) | 21.6 (14.4, 28.8) |  | 8.2 (4.9, 11.5) | 24.7 (16.3, 33.1) | 9.3 (5.5, 13.2) | 29.1 (18.3, 39.9) | 10.9 (5.9, 15.9) | 34.0 (19.5, 48.5) |
| neimeng | 2.2 (1.1, 3.2) | 6.8 (3.9, 9.8) | 3.9 (2.5, 5.3) | 11.8 (8.1, 15.6) | 6.9 (4.2, 9.6) | 20.1 (13.5, 26.6) |  | 7.7 (4.6, 10.8) | 22.7 (15.2, 30.2) | 8.7 (5.1, 12.2) | 26.2 (16.8, 35.7) | 10.0 (5.4, 14.6) | 30.3 (17.8, 42.9) |
| ningxia | 2.1 (1.1, 3.1) | 6.4 (3.6, 9.2) | 3.7 (2.4, 5.0) | 11.0 (7.5, 14.5) | 6.3 (3.8, 8.8) | 17.8 (12.0, 23.6) |  | 6.9 (4.1, 9.7) | 19.3 (12.9, 25.7) | 7.5 (4.4, 10.7) | 21.4 (13.7, 29.2) | 8.4 (4.5, 12.3) | 23.8 (13.9, 33.7) |
| qinghai | 2.1 (1.1, 3.0) | 6.3 (3.5, 9.1) | 3.5 (2.2, 4.7) | 9.9 (6.7, 13.1) | 6.0 (3.7, 8.3) | 16.0 (10.7, 21.4) |  | 6.4 (3.8, 9.0) | 16.8 (10.9, 22.6) | 6.9 (4.0, 9.8) | 18.0 (11.0, 25.0) | 7.6 (4.0, 11.2) | 19.0 (10.3, 27.8) |
| shaanxi | 2.1 (1.1, 3.1) | 6.6 (3.7, 9.5) | 3.7 (2.4, 5.0) | 11.0 (7.5, 14.5) | 6.5 (4.0, 9.1) | 18.8 (12.6, 24.9) |  | 7.2 (4.3, 10.1) | 20.8 (13.8, 27.8) | 8.0 (4.6, 11.3) | 23.7 (15.0, 32.4) | 9.1 (4.9, 13.2) | 27.0 (15.7, 38.4) |
| shandong | 2.3 (1.2, 3.4) | 7.8 (4.4, 11.2) | 4.2 (2.7, 5.7) | 14.1 (9.6, 18.6) | 7.6 (4.7, 10.5) | 23.8 (16.1, 31.6) |  | 8.7 (5.2, 12.1) | 27.8 (18.6, 36.9) | 9.9 (5.9, 14.0) | 33.2 (21.5, 45.0) | 11.7 (6.4, 16.9) | 39.3 (23.2, 55.4) |
| shanghai | 2.2 (1.2, 3.3) | 7.1 (3.9, 10.2) | 3.9 (2.5, 5.3) | 12.3 (8.4, 16.3) | 6.7 (4.1, 9.3) | 20.0 (13.5, 26.5) |  | 7.4 (4.4, 10.3) | 22.2 (14.8, 29.6) | 8.1 (4.8, 11.5) | 25.3 (16.2, 34.3) | 9.2 (5.0, 13.4) | 28.5 (16.7, 40.3) |
| shanxi | 2.2 (1.1, 3.2) | 7.0 (3.9, 10.1) | 3.9 (2.5, 5.2) | 12.1 (8.2, 15.9) | 6.5 (4.0, 9.0) | 18.2 (12.3, 24.0) |  | 7.0 (4.2, 9.8) | 19.6 (13.2, 26.0) | 7.7 (4.6, 10.8) | 21.5 (13.9, 29.2) | 8.7 (4.7, 12.6) | 23.6 (14.0, 33.2) |
| sichuan | 2.1 (1.1, 3.1) | 6.5 (3.6, 9.3) | 3.6 (2.3, 4.9) | 10.6 (7.1, 14.0) | 6.0 (3.7, 8.4) | 16.1 (10.8, 21.4) |  | 6.4 (3.8, 9.0) | 16.8 (11.2, 22.4) | 6.9 (4.0, 9.7) | 17.9 (11.3, 24.4) | 7.6 (4.1, 11.0) | 18.8 (10.9, 26.7) |
| tianjin | 2.3 (1.2, 3.3) | 7.1 (3.9, 10.2) | 4.3 (2.7, 5.8) | 14.3 (9.7, 18.8) | 7.9 (4.8, 11.0) | 24.2 (16.3, 32.1) |  | 9.1 (5.4, 12.7) | 28.3 (18.9, 37.7) | 10.5 (6.2, 14.8) | 33.8 (21.7, 45.9) | 12.4 (6.7, 18.1) | 40.1 (23.5, 56.8) |
| xinjiang | 2.1 (1.1, 3.1) | 6.6 (3.7, 9.5) | 3.7 (2.4, 5.0) | 11.7 (8.0, 15.4) | 6.6 (4.0, 9.2) | 19.6 (13.2, 26.0) |  | 7.3 (4.3, 10.3) | 22.0 (14.7, 29.4) | 8.2 (4.8, 11.6) | 25.4 (16.4, 34.5) | 9.4 (5.1, 13.7) | 29.1 (17.1, 41.2) |
| yunnan | 2.1 (1.1, 3.1) | 6.6 (3.7, 9.5) | 3.7 (2.4, 5.0) | 10.7 (7.3, 14.1) | 6.1 (3.7, 8.5) | 17.0 (11.5, 22.5) |  | 6.6 (3.9, 9.2) | 18.1 (12.0, 24.1) | 7.1 (4.2, 10.0) | 19.7 (12.6, 26.7) | 7.8 (4.2, 11.3) | 21.3 (12.4, 30.3) |
| zhejiang | 2.2 (1.1, 3.2) | 6.9 (3.9, 9.8) | 3.8 (2.4, 5.1) | 11.7 (8.0, 15.4) | 6.1 (3.8, 8.5) | 17.4 (11.7, 23.1) |  | 6.5 (3.9, 9.1) | 18.4 (12.2, 24.6) | 6.9 (4.1, 9.8) | 19.9 (12.7, 27.1) | 7.6 (4.1, 11.0) | 21.3 (12.3, 30.3) |

Figure S1 Leave-one-out analysis (A) and funnel plot (B).


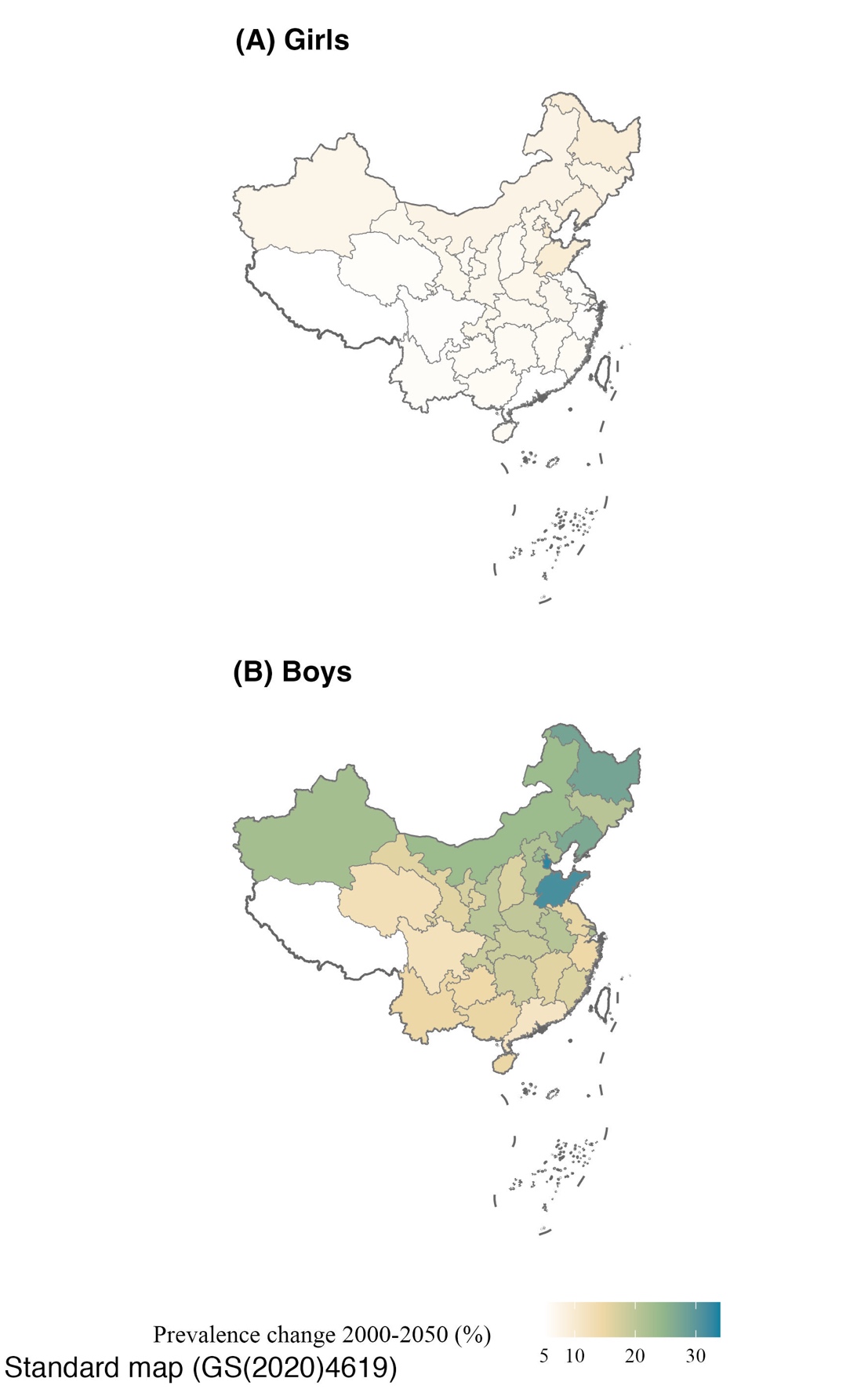


Figure S2 Increase in MASLD prevalence among children and adolescents aged 6–18 years in China from 2000 to 2050 (A): Girls; (B): Boys.

Note: MASLD, metabolic dysfunction-associated steatotic liver disease.
